# Shifts and plasticity of plant leaf mass per area and leaf size among slope aspects in a subalpine meadow

**DOI:** 10.1002/ece3.8113

**Published:** 2021-09-17

**Authors:** Xin’e Li, Xiaoyu Song, Jun Zhao, Haifeng Lu, Cheng Qian, Xin Zhao

**Affiliations:** ^1^ Division of Grassland Science College of Animal Science and Technology Yangzhou University Yangzhou China; ^2^ Northwest Institute of Eco‐Environment and Resources Chinese Academy of Sciences Lanzhou China; ^3^ Department of life sciences Lvliang University Lvliang China

**Keywords:** heat load, intraspecific trait variation, leaf morphological traits, soil properties, species turnover

## Abstract

The composition of vegetation on a slope frequently changes substantially owing to the different micro‐environments of various slope aspects. To understand how the slope aspect affects the vegetation changes, we examined the variations in leaf mass per area (LMA) and leaf size (LS) within and among populations for 66 species from 14 plots with a variety of slope aspects in a subalpine meadow. LMA is a leaf economic trait that is tightly correlated with plant physiological traits, while the LS shows a tight correlation with leaf temperature, indicating the strategy of plants to self‐adjust in different thermal and hydraulic conditions. In this study, we compared the two leaf traits between slope aspects and between functional types and explored their correlation with soil variables and heat load. Our results showed that high‐LMA, small‐leaved species were favored in south‐facing slopes, while the reverse was true in north‐facing areas. In detail, small dense‐leaved graminoids dominated the south slopes, while large thin‐leaved forbs dominated the north slopes. Soil moisture and the availability of soil P were the two most important soil factors that related to both LMA and LS, and heat load also contributed substantially. Moreover, we disentangled the relative importance of intraspecific trait variation and species turnover in the trait variation among plots and found that the intraspecific variation contributed 98% and 56% to LMA and LS variation among communities, respectively, implying a large contribution of intraspecific trait plasticity. These results indicate that LMA and LS are two essential leaf traits that affect the adaptation or acclimation of plants underlying the vegetation composition changes in different slope aspects in the subalpine meadow.

## Graphical abstract

LMA and leaf size significantly changed along a south‐to north facing slope aspect gradient indicating a leaf strategy change in different habitat. Soil moisture and the availability of soil P are the two most important factors that relate to both LMA and leaf size. Moreover, we disentangled the relative importance of intraspecific trait variation and species turnover in the trait variation among plots and found that intraspecific variation contributed 98% and 56% to the trait variation, respectively, implying a large contribution of intraspecific trait plasticity.

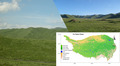

## 1 INTRODUCTION

Slope aspect is an important topographic factor that underlies the variation of vegetation composition and attributes and contributes to the substantial heterogeneity of landscape and high species diversity at the local scales (Li et al., 2011; Sidari et al., 2008; Singh, 2018; Warren, 2008; Warren II, 2010; Yang et al., 2020). For example, the previous studies have shown a higher richness and coverage of forbs species on north‐facing slopes (NFS) compared with south‐facing slopes (SFS; Li et al., 2011). To reveal the underlying mechanisms of vegetation changes in different habitats, it is critical to detect how plants adjust their functional traits, since plant functional traits are directly associated with environmental filters (Diaz et al., 1998).

Plant traits exhibit significant shifts across environment gradients, implying different growth strategies at both global and local scales. The ratio between leaf dry mass and leaf area (“Leaf Mass per Area,” LMA in g/m^2^) can be understood as the leaf level cost of light interception (Gutschick & Wiegel, 1988). LMA is a key trait in plant growth (Lambers & Poorter, 1992) and an important indicator of plant strategies (Grime, 2001; Westoby et al., 2002). A higher LMA largely correlates with a thicker cell wall (Onoda et al., 2004) and cuticles (Soh et al., 2017; Veromann‐Jürgenson et al., 2020). Moreover, in the aspect of chemical composition, high‐LMA leaves have higher concentrations of cell wall compounds and lower concentrations of cytoplasmic compounds than low‐LMA species (Mediavilla et al., 2008). Therefore, higher‐LMA species tend to have lower leaf nitrogen and phosphorus concentrations and lower photosynthetic and respiration rates per mass (Onoda & Wright, 2018; Onoda et al., 2017). Moreover, species with a higher LMA have longer leaf longevity owing to the stronger toughness of leaves (Wright et al., 2004, 2005). These correlations are summarized into a single major axis called the “leaf economics spectrum,” which runs from “quick‐return” to “slow‐return” species (Wright et al., 2004). Species with a higher LMA and a longer leaf longevity but a lower photosynthesis rate, and species with the reverse traits dominate on the slow‐ and fast‐return end, respectively (Wright et al., 2004). Poorter et al. (2009) showed that LMA was determined by both leaf thickness and density and varied strongly with light, temperature, and submergence and moderately with CO_2_ concentration and nutrient and water stress. Plants typically tend to have a higher LMA and longer leaf longevity in dry land and infertile sites (Gong & Gao, 2019; Wright et al., 2005), with the implication that plants increased their LMA to form a trade‐off between leaf longevity and photosynthetic rates, that is, the plants increased their leaf life span at the cost of photosynthesis, and the reverse is true.

Leaf temperature is another key factor that regulates the net rates of photosynthesis (Jones, 2013). Leaf size (LS; here refers to the one‐sided projected area of single leaves) affects leaf temperature via the absorbance and emission of long‐ and short‐wave incident solar irradiance. According to the energy balance theory (Parkhurst, 1971), large leaves with a higher leaf boundary layer thickness that resist the heat exchange between leaf and air are less closed to the air temperature, while smaller leaves easily approach the atmospheric temperature. Stomatal transpiration and convective heat exchange that depend on the leaf‐air temperature differentials are the two main ways of cooling for leaves in hot conditions. All the leaves are cooled by transpiration, but large leaves are more vulnerable to heat damage, particularly in water‐limiting conditions. In addition, leaves are restricted by both day and night energy balances; for example, large leaves are more vulnerable to nighttime chill damage, but they are also limited by over‐heating in hot and dry regions when transpiration is low during the day (Lambers et al., 2008). On this topic, Wright et al. (2018) reported that across the world at drier sites, LS was primarily restricted by the daytime energy balance but limited by the nighttime energy balance in wet sites. Consequently, they showed that leaves were only smaller at drier sites in warm regions, only smaller at hotter sites in dry regions, and smaller at colder sites, particularly under wetter conditions (Wright et al., 2018). Recent studies have also shown that smaller leaves lose water more quickly, which is consistent in its effective thermal regulation in high light intensity and hot environments (Wang et al., 2019). LS is also influenced by additional factors; for example, large leaves can be favored under shade (Givnish, 1987), and lower soil nutrients can also limit the LS (Carlos et al., 2000). As Givnish (1984) indicated, the leaf temperature increased with LS, which at first improved the photosynthetic rate, but as the LS continued to increase, the rate of photosynthesis decelerated since other factors rather than carboxylation limited the uptake of CO_2_. In moist but sterile sites, transpiration costs are relatively high compared with the photosynthetic benefits that accrue from a given increase in LS, so that small leaves are favored.

Few studies investigated leaf traits along the slope aspect gradient. Slope aspects may influence the leaf trait by both climate and soil factors. Ackerly et al. (2002) found higher LMA and smaller LS in the SFS in a forest of the coastal California region, and Li et al. (2019) showed that the leaves on NFS at the population level in an alpine steppe were large, but more evidence is still required to delineate the plants in different climate zones or vegetation types. As is well known, incident solar radiation is higher on SFS in the Northern Hemisphere. Significant correlations of LS and LMA with irradiance were found along the slope aspect gradient according to Ackerly et al. (2002). Probably the higher LMA helped to prevent water loss and limit the susceptibility to desiccation and the smaller LS easily conducted convective heat exchange for cooling in hot, dry SFS with high irradiance. However, as we described above, in addition to climate factors, soil nutrients also restricted the LMA and LS, but no studies have explored their correlations with soil variables along such a gradient. On the one hand, the higher irradiance in SFS results in higher air and soil temperatures (Nobel & Linton, 1997), while there is a consistent pattern that soil moisture is generally higher on NFS (Geroy et al., 2011; Nobel & Linton, 1997; Wang et al., 2011), particularly in the organic and upper soil layers (Nobel & Linton, 1997). On the other hand, vegetation can also influence soil factors. For example, the greater vegetation density in the NFS enhanced the vegetation transpiration, but also decreased soil water evaporation (Wang et al., 2011). The nurse shrub *Potentilla fruticosa* L. growing in the north‐facing slope aspects greatly increased the understory soil temperature in our study region (Xu et al., 2010). Consequently, the nutrient mineralization and availability may be altered by the different temperature and moisture on north‐ compared with south‐facing slopes (Miller & Poole, 1979). Sidari et al. (2008) demonstrated a substantial influence of slope aspects on soil chemical and biochemical properties, and Singh (2018) reported that NFS was supported with thick and dense vegetation with nutrient‐rich soil, whereas SFS had thin and scattered vegetation along with weaker soil development and higher erosion rates. In addition, although the potential incidence of direct radiation is symmetrical around the north–south axis, the heat load is symmetrical around the northeast–southwest line since a slope that receives afternoon sun will have higher maximum temperatures than an equivalent slope that receives morning sun (McCune & Keon, 2002). Therefore, the way the soil factors and heat load impact leaf traits on the slope aspect gradient merits further study.

Moreover, plants often display large intraspecific variability in functional traits, which arises from phenotypical plasticity or genetic adaptation (Jung et al., 2010), and this variation influences plant responses to abiotic filters and biotic interactions (Fridley & Grime, 2010; Fridley et al., 2007; Ravenscroft et al., 2014). Thus, the functional trait variability within a population influences its ability to respond or evolve with environmental changes (Nicotra et al., 2010). A growing number of studies have suggested that trait variability is substantial within populations (Jung et al., 2010; Laforest‐Lapointe et al., 2014; Violle et al., 2012). A global meta‐analysis found that intraspecific trait variation (ITV) contributed 32% of the total variation of 36 traits among communities and differed among different types of functional traits (Siefert et al., 2015). Species turnover is another contributor in the trait shifts among communities in addition to ITV. According to Ackerly and Cornwell (2007), the regression line slope of ITV against plot‐level trait means in a trait gradient relationship can be an indicator of the trait plasticity. By definition, if the plot trait means are caused by only the intraspecific variation, the slope should be 1. In contrast, the slope will be 0 if none of the species have plasticity, and all the shifts of plot trait means result from species turnover along the environmental gradients. To reveal the plasticity of LS and LMA is critical to understand the biotic mechanism of plant adaptation or acclimation along the slope aspect gradient.

Alpine meadow is a major vegetation component of the Qinghai–Tibet Plateau and has a short growing period and low biomass production. Plant communities are subjected to a severe plateau climate, with strong solar radiation and cold and dry conditions (Qiao & Duan, 2016). Moreover, the structure of alpine landscapes is shaped primarily by small‐scale topographic heterogeneity. Such heterogeneity in microhabitat conditions may have a sorting effect on certain plant trait values relative to individuals' tolerance to those conditions (Berend et al., 2019). Therefore, the trait plasticity is important in alpine environments. Thus, based on the slope aspect gradient in subalpine meadows, we asked the following questions: (1) How did plants shift their LMA and LS to adapt to different habitats, and was there a difference among functional groups since the dominant functional types changed in different slope aspects? (2) How did the two leaf traits correlate with soil factors and heat load? (3) To what extent did the ITV and species turnover result in plot‐level LMA and LS shifts? We aimed to detect what type of leaf strategy plants adopted in different habitats and the underling abiotic and biotic mechanisms.

## 2 MATERIAL AND METHODS

### 2.1 Study site and data collection

**FIGURE 1:**
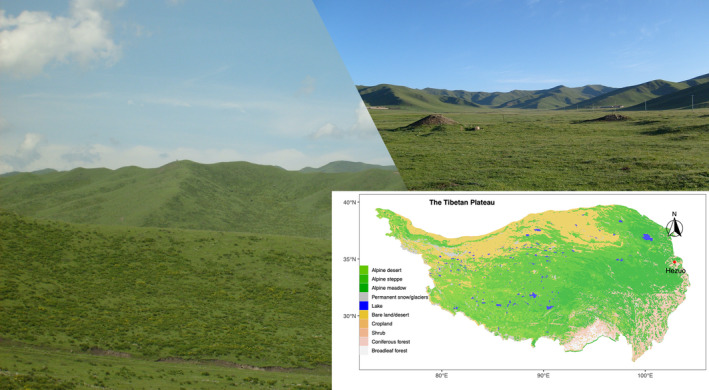
The site location (the red point) in the Tibetan plateau and the landscape in this region

Experiments were conducted in a subalpine meadow of the eastern part of the Tibetan Plateau (34̊4' N, 102̊5' E, and 2,960 m; Figure 1) near the Research Station of Alpine Meadow and Wetland Ecosystem of Lanzhou University located in Hezuo, Gansu, China. The climate is characterized by an alpine humid climate. The annual temperature averages 4ºC with maximum and minimum monthly temperature 21ºC and −13ºC in July and January, respectively, and the mean annual precipitation is 557.8 mm mainly concentrated at the intervals from May to September, according to the 1981–2017 climate data (http://data.tpdc.ac.cn).

From 2008 to 2010, we selected three hills (or sites). Based on the hill shape and availability, plots were established on five slope aspects on the western half of each hill to form a south‐to‐north gradient. In this study, "south" and "north" indicated the equator‐ and pole‐facing slope aspects, respectively, since our studies were conducted in the northern hemisphere. These slope aspects faced south, southwest, west, northwest, and north and were determined using a compass. The three hills were carefully selected to contain smoothly transitional slope aspects and similar slopes to avoid the impact of different degrees of steepness. Thus, we had 14 plots in total since one slope aspect lacked data on the traits. The three hills (sites) were approximately thousands of meters away from each other with the same elevation and similar species composition. Data collecting was conducted in July and August during the growing season. We chose nearly all species that were present to collect leaves on each slope aspect within a 5 m × 5 m plot to measure leaf traits except for a few rare species. Species were simply classified into four plant functional types (PFT): the nonlegume forbs (forbs), legumes, graminoids (sedge and grass family), and shrubs.

### 2.2 Leaf traits and soil factors measurements

We selected 10–20 leaves of 5–10 individuals for each species to measure their leaf traits, and then, the leaf trait values from within‐species individuals were averaged to represent the species‐level leaf traits. The leaf was scanned using a portable scanner (Epson, Perfection, V39, Indonesia), and then, LS (cm^2^) was calculated with the software ImageJ (NIH, Bethesda, MD, USA). Especially, some graminoid species had folded leaves and thus scanning would underestimate their LS, so we multiplied the scan area by two for these species. After scanning, the fresh leaf was dried at 70°C for 48 hr, and then, the leaf dry mass (g) was weighted. For each species, LMA (g/m^2^) was calculated as the ratio of leaf dry mass to leaf area. In total, 344 observations were collected with 64 species across 14 plots. All the species name and family were standardized according to the Taxonomic Name Resolution Service v. 4.1 (http://tnrs.iplantcollaborative.org/TNRSapp.html). The mean, maximum, and minimum leaf traits values for all the 64 species are shown in Table 1.

**TABLE 1:** All species measured across the 14 plots with their mean, maximum, and minimum LMA (Leaf mass per area) and leaf size (LS)

Species names	Family	Plant functional type	LMA (g/m^2^)			LS (cm^2^)		
			Maximum	Mean	Minimum	Maximum	Mean	Minimum
*Bupleurum* sp.	Apiaceae	Forbs	70.95	61.89	57.61	3.45	1.60	0.97
*Saposhnikovia divaricata*	Apiaceae	Forbs	80.99	80.99	80.99	3.49	3.49	3.49
*Ajania tenuifolia*	Asteraceae	Forbs	81.96	74.29	59.51	0.71	0.43	0.16
*Anaphalis* sp.	Asteraceae	Forbs	60.90	60.69	60.47	2.33	1.87	1.40
*Anaphalis lactea*	Asteraceae	Forbs	75.33	63.22	48.42	6.13	3.86	1.57
*Artemisia* sp.	Asteraceae	Forbs	49.79	48.44	47.09	2.13	1.73	1.33
*Artemisia desertorum*	Asteraceae	Forbs	81.48	71.36	60.26	3.13	1.97	0.91
*Artemisia mongolica*	Asteraceae	Forbs	47.67	47.67	47.67	1.10	1.10	1.10
*Artemisia sieversiana*	Asteraceae	Forbs	91.42	76.35	63.12	6.41	3.81	2.39
*Aster hispidus*	Asteraceae	Forbs	66.17	60.49	56.53	0.71	0.57	0.40
*Lactuca sibirica*	Asteraceae	Forbs	53.99	44.53	37.81	2.21	1.28	0.83
*Leontopodium leontopodioides*	Asteraceae	Forbs	110.67	75.92	51.18	0.85	0.46	0.14
*Picris hieracioides* L.	Asteraceae	Forbs	50.12	50.12	50.12	12.22	12.22	12.22
*Saussurea* sp.	Asteraceae	Forbs	114.93	103.34	93.08	16.23	10.73	6.38
*Saussurea eriocephala*	Asteraceae	Forbs	109.40	82.81	64.90	24.24	8.30	3.32
*Saussurea hieracioides*	Asteraceae	Forbs	91.14	83.54	76.42	9.75	7.29	4.88
*Saussurea nigrescens*	Asteraceae	Forbs	61.49	61.49	61.49	7.34	7.34	7.34
*Taraxacum mongolicum*	Asteraceae	Forbs	65.86	57.00	48.29	7.52	4.05	2.27
*Xanthopappus subacaulis*	Asteraceae	Forbs	208.16	208.16	208.16	27.45	27.45	27.45
*Parnassia oreophila*	Celastraceae	Forbs	53.72	53.72	53.72	1.24	1.24	1.24
*Crassulaceae* sp.	Crassulaceae	Forbs	60.45	60.45	60.45	0.20	0.20	0.20
*Euphorbia esula*	Euphorbiaceae	Forbs	52.18	49.20	43.19	6.01	2.62	0.22
*Euphorbia fischeriana*	Euphorbiaceae	Forbs	70.67	62.67	54.55	0.73	0.48	0.38
*Gentiana lawrencei var. farreri*	Gentianaceae	Forbs	210.05	180.49	164.33	3.85	2.62	0.84
*Gentiana macrophylla*	Gentianaceae	Forbs	125.34	106.40	84.84	9.03	5.88	2.64
*Halenia corniculata*	Gentianaceae	Forbs	47.00	36.80	29.30	1.64	0.68	0.32
*Geranium wilfordii*	Geraniaceae	Forbs	95.68	80.50	66.01	3.56	1.81	0.70
*Dracocephalum tanguticum*	Lamiaceae	Forbs	131.47	131.47	131.47	0.33	0.33	0.33
*Nepeta cataria*	Lamiaceae	Forbs	51.93	51.93	51.93	4.58	4.58	4.58
*Stachys sieboldii*	Lamiaceae	Forbs	40.34	40.34	40.34	2.59	2.59	2.59
*Euphrasia pectinata*	Orobanchaceae	Forbs	54.55	52.88	51.75	0.36	0.26	0.16
*Pedicularis* sp.	Orobanchaceae	Forbs	65.61	56.53	46.80	2.34	1.59	0.38
*Lancea tibetica*	Phrymaceae	Forbs	240.24	98.99	70.77	3.42	2.16	0.89
*Plantago asiatica*	Plantaginaceae	Forbs	63.46	54.83	45.53	9.18	3.76	2.27
*Bistorta macrophylla*	Polygonaceae	Forbs	89.98	68.75	55.04	0.77	0.59	0.39
*Persicaria vivipara*	Polygonaceae	Forbs	74.07	64.90	52.39	10.19	7.26	5.46
*Androsace erecta*	Primulaceae	Forbs	70.21	62.91	57.65	0.14	0.11	0.09
*Glaux maritima*	Primulaceae	Forbs	89.82	84.72	79.62	2.21	2.04	1.87
*Anemone obtusiloba*	Ranunculaceae	Forbs	96.20	84.58	72.49	3.46	2.35	1.55
*Anemone rivularis var. flore‐minore*	Ranunculaceae	Forbs	66.04	66.04	66.04	12.05	12.05	12.05
*Thalictrum* sp.	Ranunculaceae	Forbs	92.63	77.89	70.34	1.96	1.33	0.97
*Fragaria vesca*	Rosaceae	Forbs	73.18	64.50	59.77	4.49	2.73	1.79
*Potentilla anserina*	Rosaceae	Forbs	123.35	93.57	63.45	6.27	2.73	0.95
*Potentilla bifurca*	Rosaceae	Forbs	100.18	87.43	78.86	2.57	1.69	1.07
*Potentilla fragarioides*	Rosaceae	Forbs	101.92	83.60	70.64	4.73	2.85	1.22
*Potentilla multifida*	Rosaceae	Forbs	95.77	87.09	78.87	3.88	3.09	2.09
*Potentilla potaninii*	Rosaceae	Forbs	75.76	75.76	75.76	3.31	3.31	3.31
*Sanguisorba officinalis*	Rosaceae	Forbs	81.92	81.92	81.92	14.20	14.20	14.20
*unknown species*	unknown	Forbs	73.15	73.15	73.15	6.82	6.82	6.82
*Viola striatella*	Violaceae	Forbs	44.87	40.90	36.94	1.84	1.70	1.56
*Elymus kamoji*	Asteraceae	Graminoids	107.93	84.89	58.17	2.26	1.63	0.52
*Kobresia macrantha*	Cyperaceae	Graminoids	117.65	88.12	74.09	3.03	1.93	0.73
*Kobresia humilis*	Cyperaceae	Graminoids	129.70	104.47	71.33	0.61	0.31	0.15
*Schoenoplectus triqueter*	Cyperaceae	Graminoids	219.61	173.06	113.31	2.04	0.91	0.36
*Aristida adscensionis*	Poaceae	Graminoids	94.62	79.13	59.17	0.49	0.39	0.28
*Aristida adscensionis*	Poaceae	Graminoids	89.92	89.92	89.92	0.27	0.27	0.27
*Festuca ovina*	Poaceae	Graminoids	176.10	154.71	118.79	0.88	0.53	0.25
*Leymus secalinus*	Poaceae	Graminoids	183.27	183.27	183.27	0.77	0.77	0.77
*Astragalus* sp.	Fabaceae	Legumes	83.79	62.62	48.50	5.22	3.06	1.73
*Gueldenstaedtia*	Fabaceae	Legumes	121.07	86.22	68.35	3.71	2.59	1.40
*Medicago* sp.	Fabaceae	Legumes	69.16	54.53	48.27	0.93	0.70	0.39
*Oxytropis* sp.	Fabaceae	Legumes	77.54	67.82	59.02	2.67	1.39	0.91
*Thymus mongolicus*	Lamiaceae	shrub	68.16	62.55	54.14	0.27	0.25	0.23
*Potentilla fruticosa*	Rosaceae	shrub	111.79	88.90	75.74	0.81	0.58	0.37

The soil temperature and moisture measurement approaches were reported in detail in Li et al. (2011). In the growing season (from July to September), we measured the daily soil temperature and moisture for each slope aspect on July 9th, August 3rd, August 26th, and September 19th using a soil data collector (Em50 data logger, Decagon Devices, Pullman, WA, USA) and obtained the data over a 24‐hr period at the intervals of half an hour. As reported in Li et al. (2011), the average daily soil temperature was 22.33ºC, 21.23ºC, 20.75ºC, 18.99ºC, and 18.13ºC, and soil moisture 17%, 18%, 16%, 23%, and 29% on south‐, southwest‐, west‐, northwest‐, and north‐facing slopes, respectively. Beyond that, we also collected five soil samples from each slope and measured the soil nitrogen (N), soil phosphorus (P), and soil pH. Soil samples were collected using a 5‐cm‐diameter corer to a depth of 15 cm. The soil was first air‐dried for more than one month. The total N was then determined according to the Kjeldahl method with digestion in sulfuric acid and K_2_SO_4_: CuSO_4_: Se catalyst (Charley & West, 1975), followed by steam distillation with a VAPODEST 40 programmable distillation system (Gerhardt, Germany). The alkaline hydrolysis method was used to determine the availability of soil nitrogen (N). A colorimetric method of molybdenum blue complex was used to determine the soil total P and available P as described by Olsen et al. (1954). A ratio of 2.5:1 water to soil was used to determine the soil pH value.

### 2.3 Heat load estimation

McCune and Keon (2002) reported that the aspect was a poor variable and should be transformed in several ways depending on the environmental factor being studied. Although the potential incidence of direct radiation is symmetrical around the north‐south axis, a reasonable approximation to heat load is to ensure that the scale is symmetrical around the northeast–southwest (NE‐SW) line since a slope that receives afternoon sun will have higher maximum temperatures than an equivalent slope that receives morning sun. In this study, we used the following equation to rescale the aspect to a scale of zero to one:

$$\text{Heat load index}=1‐\text{cos}\left(\Theta ‐45\right).$$

where *Θ* was the aspect in degrees that were east of north. Finally, the heat load index was obtained with the highest value of 1 on southwest, and the lowest 0.14 on north, and then 0.85 on south and west and 0.5 on northwest slope aspects.

### 2.4 Trait gradient analysis

As described by Ackerly and Cornwell (2007) and Dong et al. (2017, 2020), the trait plasticity was measured by the slopes in the regression of within‐species trait variation against plot‐level trait mean values. If a trait is perfectly plastic, the slopes derived from all species will have a unity. The common within‐species slope in this approach is a measure of the fraction of trait variation owing to phenotypic plasticity and/or genotypic variability, that is, the extent to which the trait shift with slopes was owing to trait plasticity. Alternatively, its complement of 1 is the measure of the fraction owing to species turnover. For example, Dong et al. (2017) showed that the plasticity of LMA and leaf nitrogen content per area (*N*_area_) were both close to 0.5, implying approximately equal contributions of plasticity and species turnover to the total variation. Across our 14 plots, 66 species occurred in total, but species that occurred lower than three times were deleted. Thus, we used 44 species to calculate trait plasticity in the final analysis.

### 2.5 Data analysis

We finally got a dataset with columns: site, slope aspect, species, PFT, and traits. *First,* we tested how leaf traits were affected by slope aspects and function group treating “site” as a random factor in the mixed linear model: Traits (LMA or LS) ~ Slope Aspect (or PFT) + (1|site) using the *lmer* function in the *lme4* R package, and then conducted multiple comparisons of leaf traits for significant factors. *Second,* the correlations of leaf traits with head load index were examined. *Third,* we compared the seven soil variables between slope aspects using a one‐way ANOVA and calculated their correlation matrix, and then, we analyzed the relationship between leaf traits and soil factors using the linear and partial linear models. *Forth,* the correlation between LMA and LS was conducted at the species, functional group, and plot levels. *Finally,* the trait gradient analysis was conducted for LMA and LS, respectively, to explore their intraspecific plasticity by plotting the slope of the within‐species trait against the plot trait mean across our 14 plots and calculating the slope mean of all the regression lines. All analyses were performed with R version 4.0.3 (R Core Team, 2020) in RStudio version 1.3.1093 (RStudio Team, 2020).

## 3 RESULTS

### 3.1 Leaf traits comparison between slope aspects and between functional groups

**FIGURE 2:**
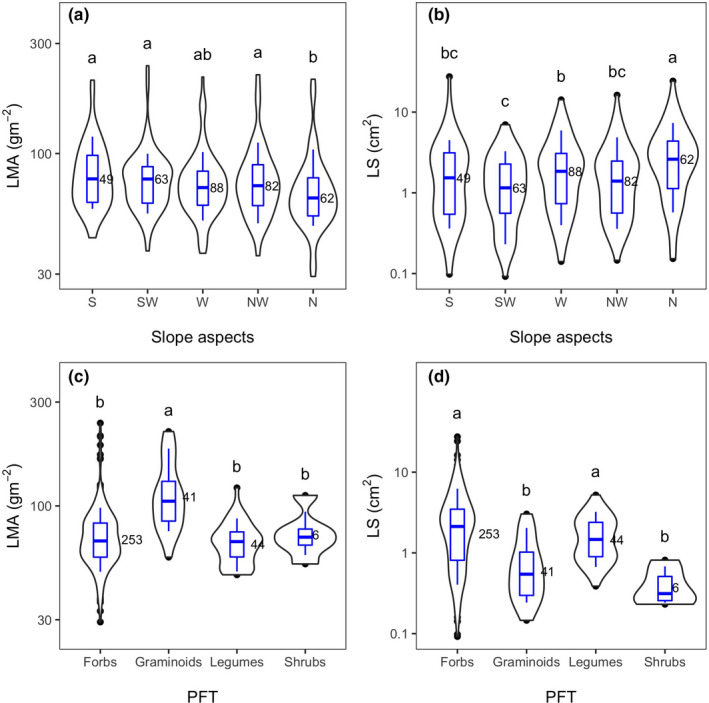
Comparisons of LMA and LS between slope aspects (panels a and b) and between plant functional types (PFT; panels c and d). The violin‐shape plot shows the density of sample distributions; boxes show interquartile ranges; and whiskers show 0.1 and 0.9 quantiles. A one‐way ANOVA and LSD were used to test the significance of multiple comparisons. Different characters indicate significant differences at the level of *p* < .05. N, north‐facing; NW, northwest; W, west‐facing; SW, southwest; and S, South‐facing slope aspects. Both LMA and LS were log‐transformed in all the figures. LMA, leaf mass per area and LS, Leaf size. The sample size was shown for each violin plot

The mean, maximum, and minimum of LMA and LS for all the 64 species were shown in Table 1. The LMA ranged from 29.30 to 240.24 g/m^2^, and the LS from 0.09 to 27.45 cm^2^ across all the observations (Table 1). The slope aspects and PFT showed significant effects on LMA and LS (Table 2). The LMA was significantly higher, but the LS was lower on SFS than on NFS (Figure 2a, b), implying that species with smaller leaves with higher LMA were favored on SFS in subalpine meadows. In addition, the LMA and LS were also significantly different among functional types: Forbs and legumes had a lower LMA and higher LS than graminoids, but shrubs showed no significant difference from the other types (Figure 2c, d).

**TABLE 2:** Effects of slope aspect and plant functional types (PFT) on leaf mass per area (LMA) and leaf size (LS). A linear mixed model was used treating site (or hill) as the random factor. LMA and LS were log‐transformed

Leaf traits	Factors	SumSq	MeanSq	NumDF	DenDF	*F* value	*p*
LMA	Slope Aspect	0.186	0.046	4	339	2.1741	.071
	PFT	1.451	0.483	3	340	27.367	<.001
LS	Slope Aspect	9.772	3.257	3	339.15	17.765	<.001
	PFT	3.749	0.937	4	318.36	4.6221	.001

**FIGURE 3:**
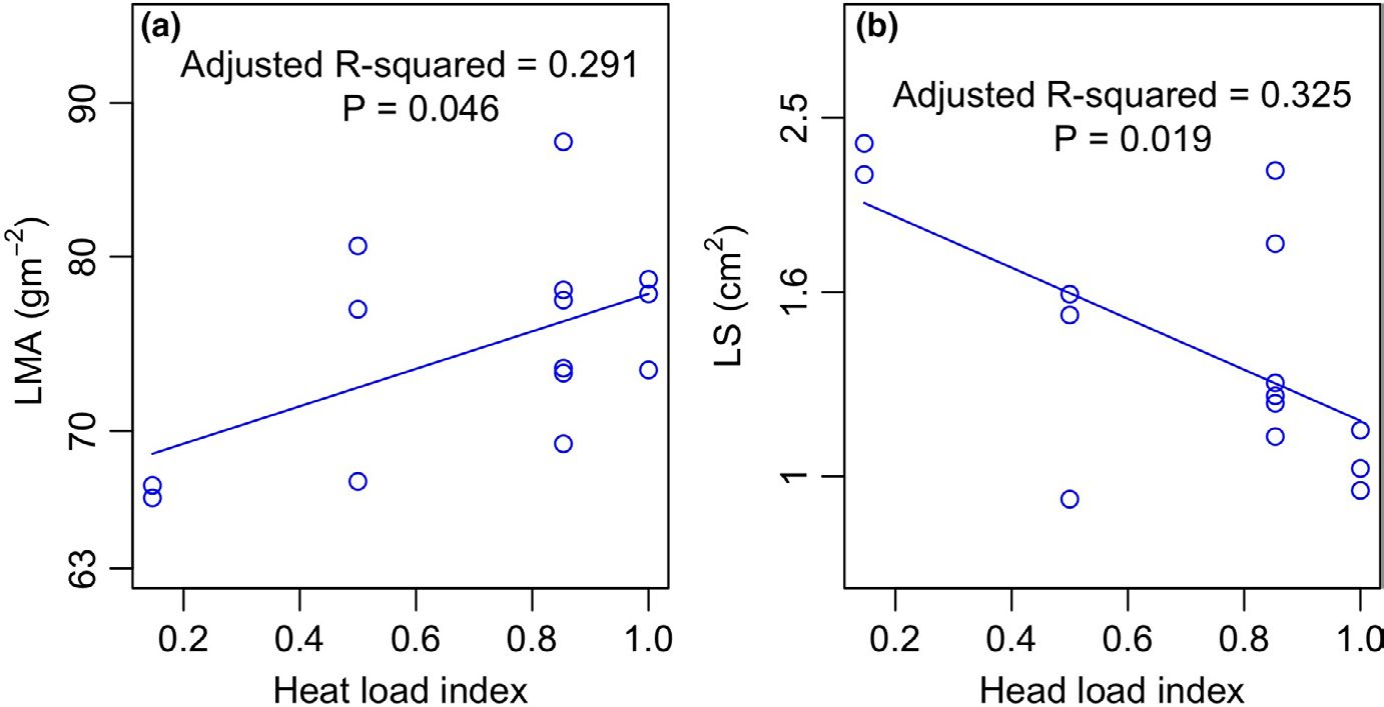
Linear regression of LMA (a) and LS (b) with heat load index. LMA, leaf mass per area and LS, leaf size

Moreover, based on the transformation of slope aspects, the correlation of leaf traits with the heat load index was measured. The LMA significantly positively correlated with the heat load index (*R* ^2^ = 0.291, *p* = .046; Figure 3a), while the LS negatively correlated with it (*R* ^2^ = 0.325, *p* = .019; Figure 3b).

### 3.2 Effects of soil variables on the LMA and LS

**FIGURE 4:**
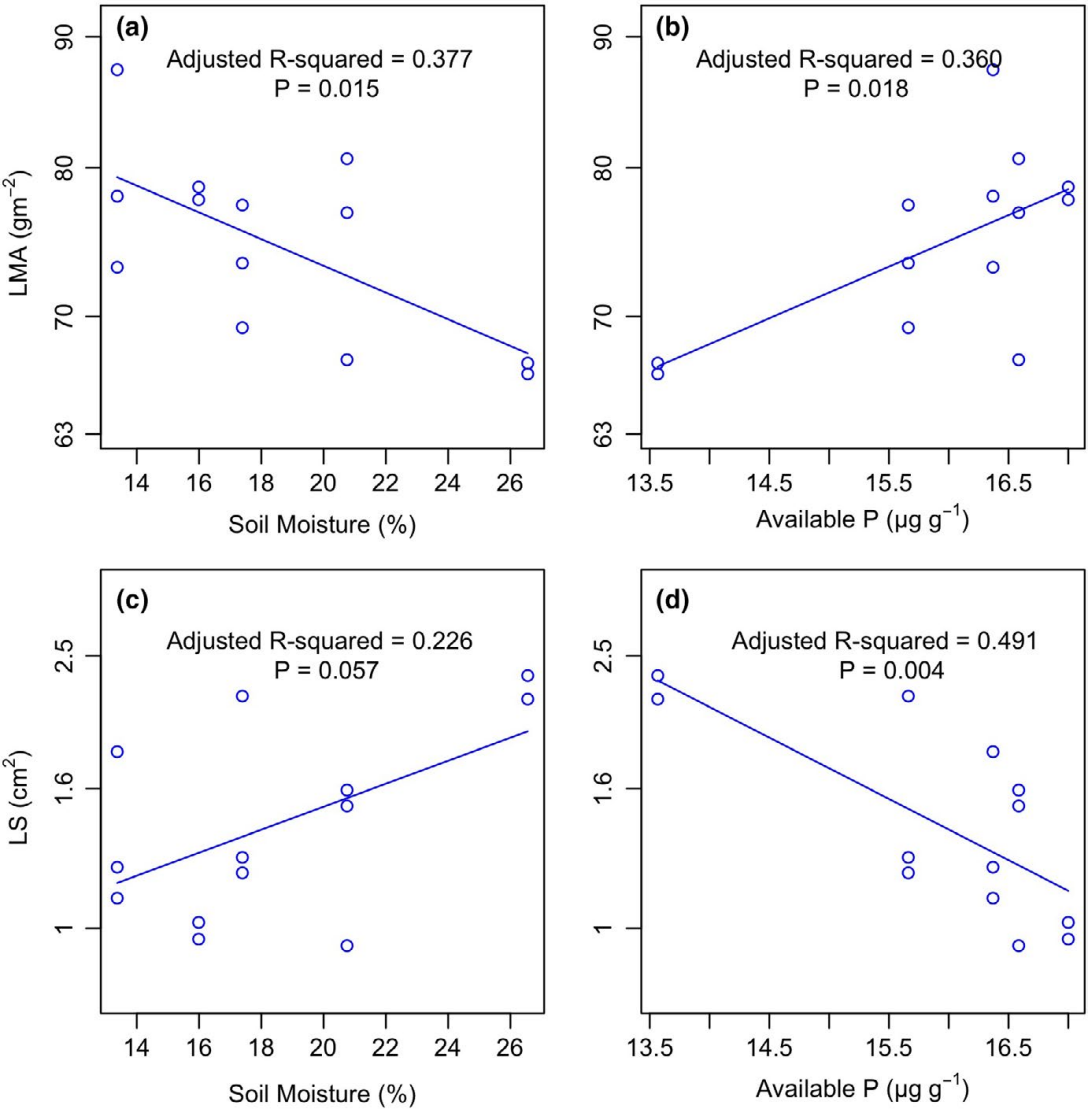
Linear regressions of LMA–soil moisture (a), LMA–soil available phosphorus (b), LS–soil moisture (c), and LS–soil available phosphorus (d) at the plot level. LMA, leaf mass per area and LS, leaf size. *N* = 14

A comparison of soil variables showed that the highest total and available nitrogen, but the lowest total phosphorus, occurred on SFS, and the available phosphorus showed no significant difference among slopes; the pH was higher on both SFS and NFS and lower in between (Table 3). Among all seven soil variables (plus the soil temperature and moisture), the LMA was significantly negatively correlated with soil moisture (*R* ^2^ = 0.377, *p* = .015; Figure 4a) and positively with the soil available phosphorus (*R* ^2^ = 0.360, *p* = .018; Figure 4b). In addition, LMA was also associated with soil temperature (*R* ^2^ = 0.289, *p* = .03) and soil total phosphorus (*R* ^2^ = 0.316, *p* = .026) in a bivariate correlation (data not shown). However, in the correlation matrix of soil variables (Table 4), the strongest correlation occurred among soil moisture, temperature, and total phosphorus with all *R* ^2^ > 0.79 (Table 4). To eliminate their covariation effects, partial analysis was done. When controlling the soil temperature, the soil moisture was still significantly associated with LMA, but the soil temperature was no longer significant when controlling soil moisture. The effects of total P became insignificant when either soil moisture or soil temperature was controlled (data not shown). LS was positively correlated with soil moisture (*R* ^2^ = 0.226, *p* = .057; Figure 4c) and negatively with soil available P (*R* ^2^ = 0.491, *p* = .004; Figure 4d).

**TABLE 3:** Differences of soil properties between slope aspects (S, South; SW, Southwest; W, West; NW, Northwest; and N, North)

Slopes	TN (mg/g)	TP (mg/g)	AN (μg/g)	AP (μg/g)	pH
S	5.34 ± 0.25 a	0.52 ± 0.01 c	45.39 ± 5.58 a	16.37 ± 3.15 a	7.83 ± 0.07 a
SW	5.52 ± 0.39 a	0.59 ± 0.02 b	42.49 ± 5.46 ab	17.00 ± 4.28 a	6.77 ± 0.12 b
W	3.08 ± 0.05 d	0.61 ± 0.08 ab	34.44 ± 9.94 bc	15.66 ± 2.49 a	6.99 ± 0.32 b
NW	3.66 ± 0.18 c	0.62 ± 0.05 ab	27.09 ± 0.79 c	16.59 ± 2.72 a	6.82 ± 0.15 b
N	4.29 ± 0.08 b	0.66 ± 0.04 a	41.32 ± 9.63 ab	13.57 ± 1.62 a	7.98 ± 0.14 a

Note

Different characters represent significant differences at the level of *p* < .05. The one‐way ANOVA and LSD multicomparison method were used to test the significance.

Abbreviations: AN, available nitrogen; AP, available phosphorus; TN, total nitrogen; TP, total phosphorus.

**TABLE 4:** The correlation matrix of soil abiotic variables: soil moisture (SM), soil temperature (ST), total nitrogen (TN), TP (total phosphorus), available nitrogen (AN), available phosphorus (AP), and pH values

	SM (%)	ST (℃)	TN (mg/g)	TP (mg/g)	AP (μg/g)	AN (μg/g)
SM (%)	1					
ST (℃)	−0.97 (***)					
TN (mg/g)	−0.42	0.51				
TP (mg/g)	0.89 (***)	−0.9 (***)	−0.61 (*)			
AP (μg/g)	−0.74 (**)	0.56 (*)	0.28	−0.57 (*)		
AN (μg/g)	−0.38	0.58 (*)	0.79 (**)	−0.53	−0.15	
pH	0.11	0.08	0.39	−0.26	−0.61 (*)	0.67 (*)

Note

*p < .05. **p < .01. ***p < .001.

### 3.3 Correlation of LMA with LS at the species, functional group, and plot levels

**FIGURE 5:**
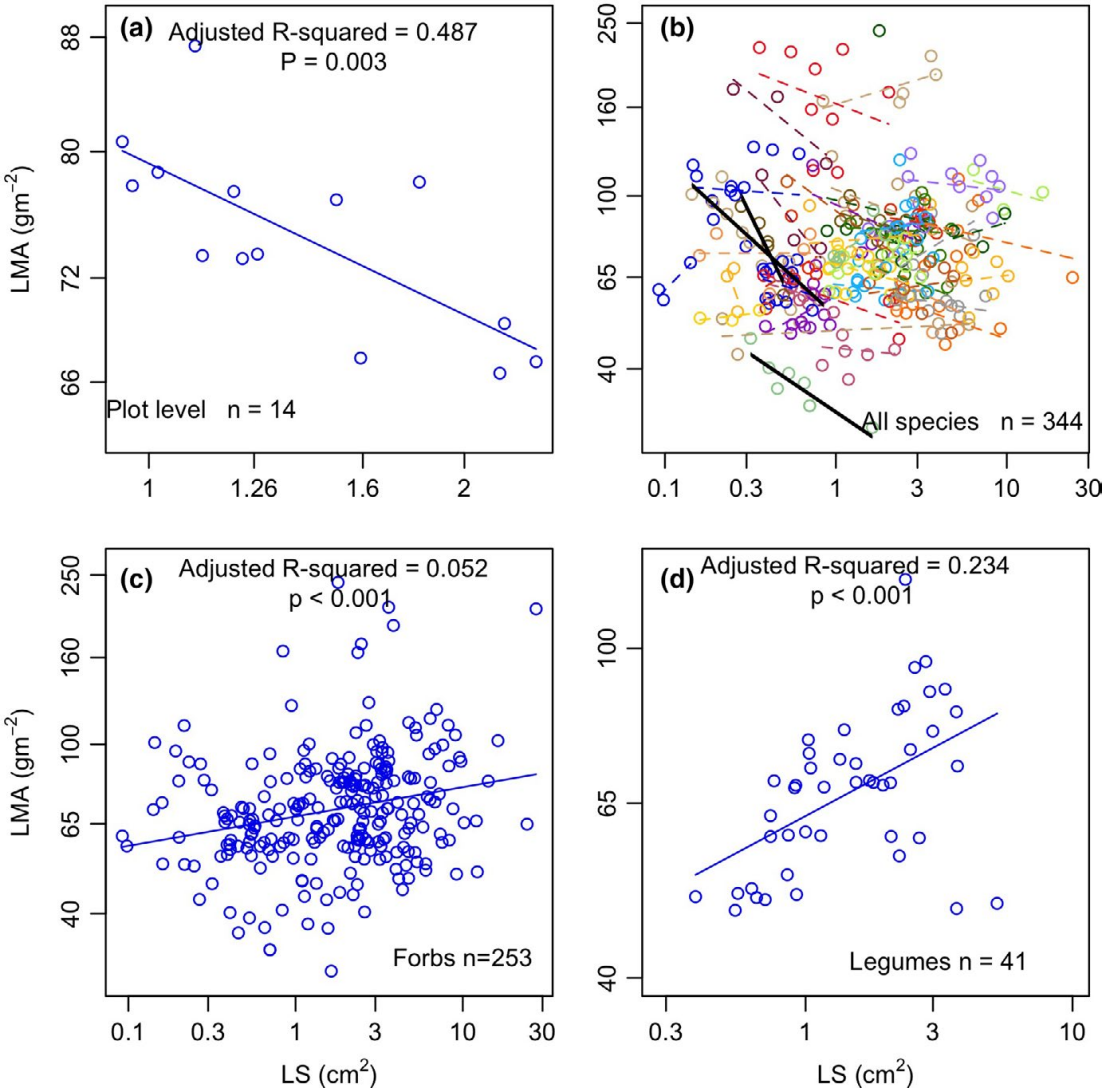
Linear relationships between LMA (leaf mass per area) and LS (leaf size) at the plot level (a), at cross and within species level (b) and the functional group level: forbs (c), and legumes (d). Regression analyses for graminoids and shrubs are not shown, since they are not significant. In panel b, the three bold black lines represent the species with significant correlations, and other species are not significantly correlated with the dashed lines

The LMA and LS were negatively correlated at the plot level (*R* ^2^ = 0.487, *p* = .003; Figure 5a) but not significantly correlated across the species (Figure 5b). At the within‐species level, only 3 species showed significant correlations (Figure 5b). However, if all the species were divided into four functional groups, positive correlations were significant within the forbs (*R* ^2^ = 0.052, *p* < .001; Figure 5c) and legumes (*R* ^2^ = 0.234, *p* < .001; Figure 5d). The correlations for the graminoids and shrubs were not significant (data not shown).

### 3.4 Intraspecific trait plasticity

**FIGURE 6:**
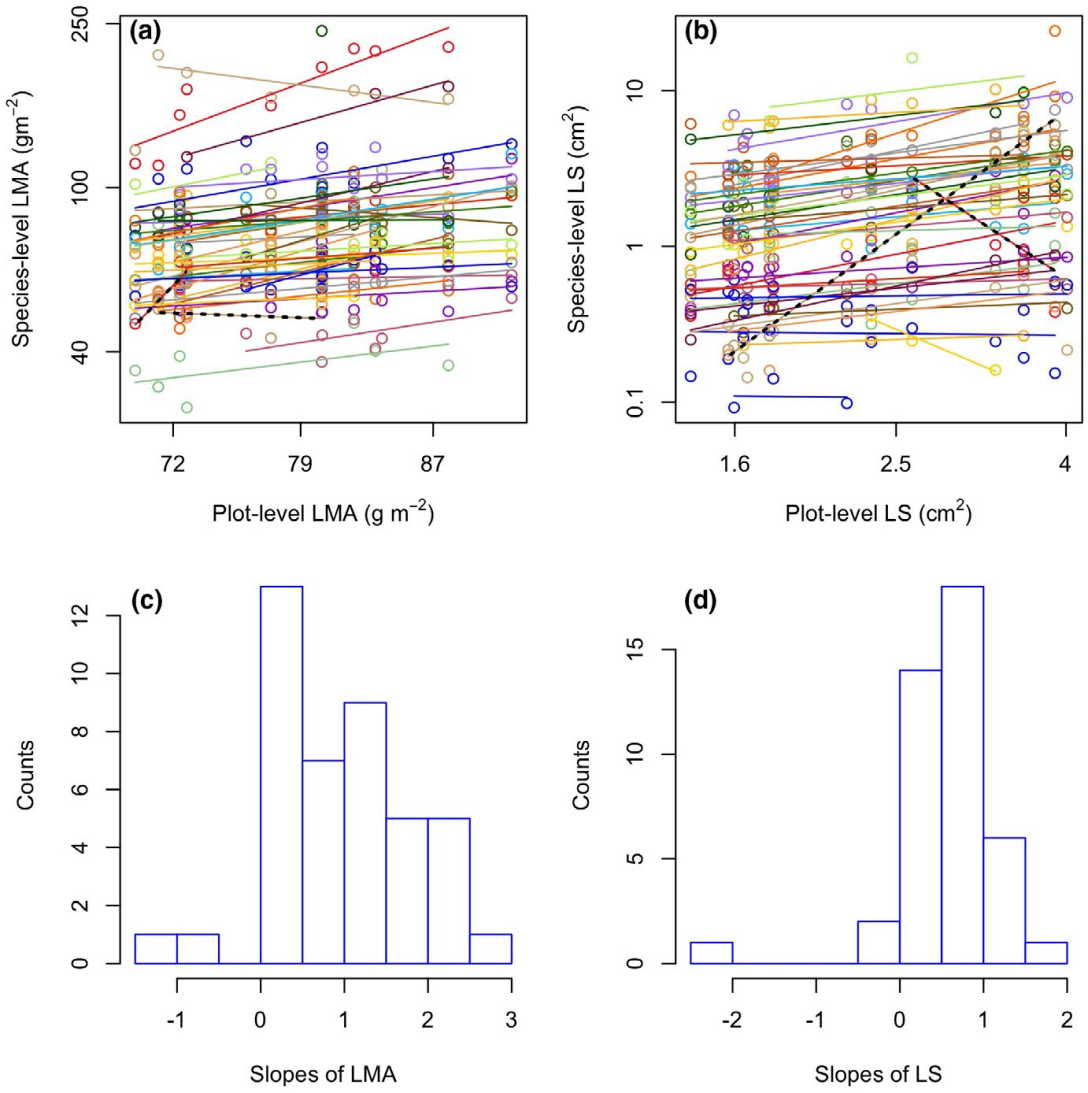
Linear regressions between within‐in species traits value and plot mean trait across 14 plots for LMA (a) and LS (b). Different colors represent regressions of different species. The two black color lines represent the two species with extremely higher or lower slopes out of a general slope range (see the main text). The histogram distributions of regression slopes in panels (a) and (b) are shown in panels (c) and (d) without the two species showing extreme slopes

Across the 44 species with more than three occurrences in our survey, trait gradient analysis for each species was conducted with within‐species variation against plot means to fit the regression slopes. Most of the slopes for LMA ranged from −1.00 to 2.88, and the LS ranged from −2.26 to 1.73 except for two species, *Pedicularis flava* Pall. and *Euphorbia* *esula* L. Strangely, the slope of *P*. *flava* was much higher than those of all the other species, with a slope of 8.0 for the LMA and a LS of 2.88 (Figure 6a), while the LMA slope for *E*. *esula* was essentially the lowest with a value of −3.50 (Figure 6b). Therefore, these two species were removed during the slope mean calculation to achieve a more reasonable value. The average slope means between the within‐species LMA variation and plot mean LMA was 0.98, while for the leaf size, the slope mean was 0.56 (Figure 6), which indicated that intraspecific variation contributed 98% and 56%, while species turnover had a lower contribution of 2% and 44% to the LMA and LS plot‐level variation, respectively.

## 4 DISCUSSION

In this study, our results showed a clear shift of plot‐level LS and LMA from SFS to NFS, which provided evidence that plants adopted different leaf strategies to survive and grow in subalpine meadows. Moreover, we also clarified the process of leaf traits changing through intraspecific plasticity and species turnover. Clarifying this pattern is essential to understand the vegetation composition difference forced by slope topography and conduct management in artificial plant ecosystems in future.

### 4.1 Shifts of LMA along the south–north slope aspect gradient

Ackerly and Cornwell (2007) reported that plants were characterized by high LMA and small leaves in SFS. However, their study focused on woody species in a forest, while we focused on herbaceous species in a grassland ecosystem. Nevertheless, we also found a significant increase in LMA (Figure 2a) and a decrease in LS (Figure 2b) in SFS. In conjunction with our previous study that forbs dominated NFS and graminoids SFS (Li et al., 2011), we also compared the leaf traits of four functional groups and found that graminoids had a higher LMA and smaller leaves than forbs and legumes (Figure 2c, d); that is, SFS were occupied by small dense‐leaved graminoids and NFS by thin large‐leaved forbs.

Ackerly and Cornwell (2007) discussed the effects of irradiance on LMA, since higher LMA was coupled with thicker cuticles and thus had advantages in preventing water loss and the susceptibility to desiccation in hot, dry south slopes. However, they did not measure soil factors and were unable to detect the effects of soils (Ackerly & Cornwell, 2007). By measuring the variables of seven soils, our results showed that the LMA strongly correlated with soil moisture and soil available P (Figure 4). According to the leaf economic spectrum, the increase in LMA (dry mass per area) is associated with higher fractions of the cell wall (Onoda et al., 2004) and thicker cuticles (Soh et al., 2017; Veromann‐Jürgenson et al., 2020) rather than photosynthetic materials, and thus lowered the loss of water in dry sites with the benefit of extending their lifespan. Nevertheless, the effect of soil available P was totally opposite to the effect of soil water on LMA (Figure 4b). We expected that there would also be an increase of LMA with the lack of soil available P because plants would adopt a slow‐investment strategy of growth by enhancing the leaf lifespan in low nutrient sites according to the leaf economic spectrum (Wright et al., 2004). Surprisingly, the LMA was positively related to soil P availability (Figure 4b). The possible explanation could be the results from Mo et al. (2020). In their study, the addition of P increased the LMA through conversion of soluble sugars into the leaf tissue to enhance leaf expansion, which was confirmed by the increased structural P fraction under the addition of P (Mo et al., 2020) since phosphorus is an important component of photosynthetic complexes and cellular structures (Marschner, 2011).

### 4.2 Shifts of LS along the south–north slope aspect gradient

The LS significantly increased in the NFS in our study (Figure 2b) in accordance with other studies (Ackerly et al., 2002; Li et al., 2019). Ackerly et al. (2002) achieved a significant relationship of LS with irradiance along the slope gradient and suggested that the leaf temperature may be a constraining factor on SFS. As described in the Introduction, smaller leaves are coupled with thinner leaf boundary layers and have a higher leaf boundary conductance to improve the leaf heat exchange in high irradiance sites and dry sites (Lambers et al., 2008). In SFS, the leaf temperature increased rapidly with high irradiance. Therefore, a smaller leaf is favored to conduct rapid heat exchange. In this situation, smaller leaves can also provide protection from water loss by transpiration in this hot and dry slope aspect; in another word, larger leaves would be transpiring more and thus drawing down soil moisture more rapidly which would then cause plant to be severely stressed by drought. Therefore, we found that soil water content is the limiting factor of the LS (Figure 4c). In the NFS, the LS was not constrained due to the higher soil water content, and another likely advantage of having large leaves in the NFS was that the wide leaf‐to‐air difference may make the leaves rapidly warm up in the cool morning to obtain a quick photosynthetic return as indicated by Wright et al. (2018).

Again, the effect of available P was unexpectedly negative on LS (Figure 4d), in contrast to the soil nutrient limitation effects on LS (Carlos et al., 2000; Givnish, 1984, 1987). As we have discussed soil P availability increased LMA, the decrease of LS may reflect a trade‐off with LMA or leaf thickness. Another interesting thing is that the soil N was not a limiting factor in our study, probably because soil N can be accumulated from the atmosphere via biological fixation or deposition, but soil P is mostly derived from rock weathering (Vitousek et al., 2010) and, thus, is a better measure of the whole soil nutrient status (Carlos et al., 2000).

### 4.3 Effects of heat load on the LMA and LS along the NE‐SW slope aspect gradient

Although we found a shift in the LMA and LS from south‐ to north‐facing slopes (Figure 2), interestingly, our results also showed that the southwest aspect had a similar LMA or an even more extreme LS than the south aspect (Figure 2). As we described above, the temperature in northeast‐southwest (NE‐SW) axis is symmetrical; that is, the southwest and northeast were the warmest and coldest slope aspects, respectively. This occurred because the afternoon sunlight has a larger impact on maximum temperatures than the morning sunlight (McCune & Keon, 2002). Therefore, we also examined the correlation of leaf traits with the heat load index. As expected, the LS significantly negatively correlated with the heat load index (Figure 3b), confirming that smaller leaves were favored in hot, dry sites since the thinner boundary layer had a rapid heat exchange for cooling (Wright et al., 2018). The positive correlation between LMA and heat load also occurred (Figure 3a) because a higher LMA helped to prevent water loss in hot, dry slopes as discussed by Ackerly and Cornwell (2007).

### 4.4 Correlations between LMA and LS at different levels

The plot‐level LS and LMA were significantly negatively associated (Figure 5a); however, the nonsignificant species‐level correlation (Figure 5b) indicated that the opposite shifting of LMA and LS along the slope gradient was not forced by their developmental processes. At the species level, LS and LMA were decoupled, which has been reported in several studies (Ackerly & Cornwell, 2007; Ackerly et al., 2002; Carlos et al., 2000). The LMA was strongly correlated with leaf nitrogen content, leaf life span, and leaf photosynthetic rate according to the “slow‐fast” investment strategy. However, all these economic traits only showed weak or no correlations with other traits, such as the LS, because they had different variation partitioning at different scales (Messier et al., 2017). The variation in LS was predominantly driven by genetic differences, while the variation in the LMA was mostly explained by conspecific individuals and environmental gradients (Messier et al., 2017). The substantially different variation drivers explained why the LS and LMA were not significantly correlated. Moreover, we even found a positive correlation of LS and LMA for forbs and legumes (Figure 5c, d), which was totally opposite to the plot‐level correlation direction. The positive relationship raised the possibility that large‐leaved forbs species required tougher leaves for mechanical support. Therefore, the plot‐level correlation of LS and LMA (Figure 5b) was largely attributed to their adaptation or acclimation to environments. In addition, the nonsignificant LMA‐LS correlation for shrubs might be partly because of the small sample size.

### 4.5 Plasticity of LMA and LS among different slope aspects

In the trait gradient analysis, most within‐species slope values were in the range from zero to unity. However, in special situations, the slope values less than zero and greater than unity. According to Dong et al. (2020), these situations signified trends opposite to the community mean and indicated “over‐reaction,” respectively, which could also occur but uncommon.

Our results showed that the intraspecific plasticity of LMA and LS were 0.98 and 0.56, respectively, indicating that 98% of the plot‐level shift of LMA and 56% of the LS were owing to intraspecific variation, and 2% and 44% of LMA and LS shifts were, respectively, owing to the turnover of species along this gradient. The less importance of intraspecific variation of LS compared with LMA implied a relatively constant status of LS within species in response to different environments. Our results were consistent with Siefert et al. (2015), which also reported a higher intraspecific variation in leaf economic traits and a weaker one in traits related with leaf size. This result can also be explained by variation partitioning of LMA and LS as Messier et al. (2017) reported that the variation of LMA was largely owing to conspecific individuals and environmental gradients, but LS was owing to genetic differences. Moreover, the plasticity of LMA (0.98) and LS (0.56) in our study region was larger than that of Dong et al. (2020) with the value of 0.65 and 0.08, respectively, which was possibly owing to a more flexible response of plant to the harsh environmental conditions in the Tibetan alpine region for species adaptation or acclimation.

## 5 CONCLUSION

In conclusion, in SFS, lower LS and higher LMA were favored, with the opposite in NFS. Soil moisture and P availability were the two most predictive soil factors, emphasizing the importance of soil P in the subalpine region. The heat load was also a substantial contributor to the determination of the LMA and leaf size. Moreover, our dataset also provided strong evidence of the substantial influence of intraspecific trait variation on plant trait shifts among communities. In addition, through comparing the dominance and leaf traits of four functional groups, we found small dense‐leaved graminoids and thin large‐leaved forbs dominant in the SFS and NFS, respectively.

## CONFLICT OF INTEREST

The authors have no relevant financial or nonfinancial interests to disclose.

## AUTHOR CONTRIBUTION

**Xine Li:** Conceptualization (lead); Data curation (lead); Formal analysis (lead); Funding acquisition (lead); Investigation (lead); Methodology (lead); Project administration (lead); Resources (lead); Software (lead); Supervision (equal); Validation (lead); Visualization (lead); Writing‐original draft (lead); Writing‐review & editing (lead). **Xiaoyu Song:** Conceptualization (supporting); Investigation (equal); Project administration (supporting); Writing‐original draft (supporting). **Jun Zhao:** Conceptualization (supporting); Investigation (supporting); Writing‐review & editing (supporting). **Haifeng Lu:** Data curation (supporting); Visualization (supporting). **Cheng Qian:** Data curation (supporting); Writing‐review & editing (supporting). **Xin Zhao:** Data curation (supporting); Writing‐review & editing (supporting).

## Data Availability

The leaf trait dataset is deposited in the Dryad repository‐https://doi.org/10.5061/dryad.w6m905qq8.
